# Pyolysin of *Trueperella pyogenes* Induces Pyroptosis and IL-1β Release in Murine Macrophages Through Potassium/NLRP3/Caspase-1/Gasdermin D Pathway

**DOI:** 10.3389/fimmu.2022.832458

**Published:** 2022-03-15

**Authors:** Hongmin Liang, Bing Wang, Junwei Wang, Bo Ma, Wenlong Zhang

**Affiliations:** ^1^ Laboratory of Veterinary Immunology, Department of Preventive Veterinary Medicine, College of Veterinary Medicine, Northeast Agricultural University, Harbin, China; ^2^ Northeastern Science Inspection Station, China Ministry of Agriculture Key Laboratory of Animal Pathogen Biology, Harbin, China

**Keywords:** pyolysin, *Trueperella pyogenes*, gasdermin D, NLRP3, caspase-1

## Abstract

*Trueperella pyogenes* (*T. pyogenes*) is a commensal and an opportunistic pathogen of animals. This organism can cause inflammatory diseases, such as pneumonia, mastitis and endometritis in hosts. However, the molecular basis for the pro-inflammatory properties of this organism is still largely unknown. In the current study, using murine macrophages as model, the ability of *T. pyogenes* to induce pyroptosis was first determined. Then, pyolysin (PLO), a cholesterol-dependent cytolysin secreted by *T. pyogenes*, was found to be closely related to *T. pyogenes*-induced pyroptosis. Next, our work showed that PLO can form pores in the cell membrane, leading to the efflux of potassium (K^+^), NOD-like receptor family pyrin domain containing 3 (NLRP3) inflammasome-mediated caspase-1 activation, and gasdermin D (GSDMD) cleavage. Inhibition of the K^+^/NLRP3/caspase-1/GSDMD pathway abolished *T. pyogenes* and PLO-induced IL-1β release. Taken together, these results indicate *T. pyogenes*-induced inflammation is related to PLO-induced pyroptosis and IL-1β release. Our work shed light on the pathogenesis of *T. pyogenes* and the interaction between *T. pyogenes* and hosts’ immune system.

## Introduction

*Trueperella pyogenes* (*T. pyogenes*) is a commensal and an opportunistic pathogen of many animal species (1). This organism can cause purulent infections in animals when the host’s immune system is compromised by unfavorable factors, such as heat, wound, transportation, and primary infections (2). Cases of human *T. pyogenes* infection have been reported (3–5). Inflammatory diseases, such as pneumonia, mastitis, endometritis, and soft tissue and organ abscesses, are the main manifestation of *T. pyogenes* infection in animals (1). However, the molecular basis for the pro-inflammatory properties of this organism is still largely unknown.

*T. pyogenes* secretes a type of hemolysin called pyolysin (PLO) (1). PLO is one of the primary virulent factors of this bacterium. PLO belongs to the cholesterol-dependent cytolysin (CDC) family (6). CDC family members form pores in the cholesterol-containing membrane and are therefore classified as pore-forming toxins (PFTs) (7). One of our previous studies showed that intramuscular inoculation of recombinant PLO protein can lead to the upregulation of the expression of the proinflammatory cytokine, IL-1β, in mice (8). This finding indicates that PLO plays an essential role in the occurrence of *T. pyogenes*-related inflammation.

Pyroptosis is a type of programmed cell death (9, 10). Gasdermin D (GSDMD) is one of the executioners of pyroptosis (11). In mice, activated caspase-1 (canonical pathway) and caspase-11 (noncanonical pathway) can cleave GSDMD into two fragments (11, 12). The amino-terminal fragment of cleaved GSDMD (GSDMD-NT) can form pores on the plasma membrane and therefore causes cell lysis (13). The pore formed by GSDMD-NT is an important way for the release of pro-inflammatory cytokines (such as IL-1β and IL-18) in living cells (14). Pyroptosis also leads to the release of pro-inflammatory cytoplasmic components (9, 10). Therefore, pyroptosis is closely related to inflammation.

Challenge with bacterial pathogens may result in the cleavage of GSDMD in animals and cultured cells (15–17). Although the relationship between the bacterial pathogen that produces CDC and the lysis of the host GSDMD has not been reported, previous studies have shown that pneumolysin (PLY) secreted by *Streptococcus pneumoniae* can induce caspase-1 activation and IL-1β secretion in murine microglia and neutrophils (18, 19). These results indicate that PLY may have the ability to cause caspase-1-mediated GSDMD cleavage. PLO and PLY are members of the CDC family, and all members of the CDC family have similar structures and functions (7); therefore, we speculated that PLO may have the ability to promote inflammation by causing pyroptosis.

In the current study, we found that *T. pyogenes* could induce GSDMD cleavage in murine macrophage cells (J774A.1 cell line) by secreting PLO. Mechanistically, PLO could induce pyroptosis *via* the classical pathway, during which caspase-1 was activated through the potassium (K^+^)/NOD-like receptor family pyrin domain containing 3 (NLRP3) pathway and mediated GSDMD cleavage. The inhibition of GSDMD cleavage attenuated the IL-1β production induced by *T. pyogenes* or PLO. Our work partially explains why *T. pyogenes* exhibits pro-inflammatory properties.

## Materials and Methods

### Cells, Bacteria Strains, Antibodies and Chemicals

J774A.1 cells, a mouse monocyte/macrophage cell line, were propagated in RPMI-1640 medium (Gibco) supplemented with 10% fetal bovine serum (FBS) at 37°C with 5% CO_2_.

*T. pyogenes* (strain 0912) was cultured in Martin broth medium with 10% fetal bovine serum under aerobic conditions.

Primary antibodies included rabbit monoclonal anti-GSDMD (ab209845, Abcam), rat monoclonal anti-caspase-1(BL-645102, Biolegend), rabbit monoclonal anti-caspase-11 (ab180673, Abcam), mouse monoclonal anti-NLRP3(AG-20B-0014, Adipogen), and mouse anti-GAPDH antibody (GTX627408, GeneTex). Secondary antibodies included horseradish peroxidase (HRP)-conjugated goat anti-mouse IgG (ZB-2305, ZSGB-BIO), HRP-conjugated goat anti-rabbit IgG (ZB-2301, ZSGB-BIO) and HRP-conjugated goat anti-rat IgG secondary antibody (ZB-2307, ZSGB-BIO).

Chemicals included VX-765 (Capase-1 inhibitor) (S2228, Selleck), MCC950 (NLRP3 inhibitor) (S8930, Selleck), Necrostatin-1 (Nec-1) (receptor-interacting serine/threonine protein kinase 1 [RIP1] inhibitor) (A4213, APExBIO), Necrosulfonamide (NSA) (mixed lineage kinase domain-like protein [MLKL] and GSDMD inhibitor) (B7731, APExBIO), and Wedelolactone (Wed) (caspase-11 and NLRP3 inhibitor) (HY-N0551, MedChemExpress).

### Preparation and Characterization of Recombinant Proteins

pET-30a(+)-plo and pET-30a(+)-plo-D238R plasmids were constructed in our previous works (8, 20).

According to another previous work, the recombinant plasmid pET-30a(+)-plo W497F was constructed from pET-30a(+)-plo by polymerase chain reaction-mediated DNA mutation method (21). pET-30a(+)-plo W497F encodes rPLO W497F (named as His-PLO F497 in Billington’s work), in which the tryptophan (W) at position 497 was replaced by phenylalanine (F).

The pET-30a(+)-plo, pET-30a(+)-plo-D238R, and pET-30a(+)-plo-W497F plasmids were separately transformed into *Escherichia coli* Rosetta (DE3)™ competent cells. The *E. coli* cells were grown in Luria–Bertani medium to an optical density (600 nm) of 0.4–0.6. Isopropyl β-D-1-thiogalactopyranoside was added to the culture to a final concentration of 1 mM. The culture was further incubated at 37°C for 4 h to express the recombinant protein. The *E. coli* cells were then collected by centrifugation. The pellet was resuspended in 0.05 M phosphate buffer saline (PBS, pH 7.0) and lysed by sonication. The recombinant proteins with six histidine tags were purified from the lysate using nickel-charged resin (GE Healthcare) according to the manufacturer’s instructions. The proteins were dialyzed against PBS with 5% glycerol at 4°C for 48 h. The purified proteins (named rPLO, rPLOD238R, and rPLOW497F, respectively) were quantified by bicinchoninic acid (BCA) assay and stored at −60°C.

The hemolytic activity of the proteins was determined according to a previous work (20). In short, the recombinant protein was first adjusted to a concentration of 100 μg/mL and then diluted by serial two-fold dilution method. The diluted protein solution was mixed with an equal volume of 2% sheep red blood cells (sRBCs) in a V-bottomed 96-well microtiter plate. The mixture was incubated for 30 min at 37°C and then observed.

The cell membrane binding and pore-forming abilities of the recombinant proteins were determined according to the previous work (8).

### Preparation of Inactivated *T. pyogenes*

*T. pyogenes* (strain 0912) was cultured in Martin broth medium containing 10% fetal bovine serum (FBS) under aerobic conditions until the late-log phase. The density of *T. pyogenes* cells was determined by colony counting method. Formaldehyde was added to the culture to a final concentration of 0.5%. The mixture was incubated at 37°C for 48 h to inactivate *T. pyogenes*. Then, the inactivated *T. pyogenes* was washed twice with ice-cold sterile PBS, resuspended in sterile PBS, and stored at −80°C until use.

To determine the working concentration of the inactivated *T. pyogenes*, 10 MOI of *T. pyogenes* was incubated with J774A.1 cells (2×10^6^ cells) at 37°C for 4 h. Gently shake the container to fully suspend the bacterial cells. The density of *T. pyogenes* cells in the culture medium was determined by colony counting method and was used as the working concentration of the inactivated *T. pyogenes*.

### Lactate Dehydrogenase (LDH) Release Measurement

J774A.1 cells were seeded on a 96-well cell culture plate at a density of 1×10^4^ cells/well. The cells were allowed to grow overnight and then treated with different concentrations of rPLO and chemicals for different times. The culture supernatant was collected. The LDH release of the cells was determined using the LDH Cytotoxicity Assay Kit (C0017, Beyotime) according to the manufacturer’s instruction.

### Determination of Intracellular K^+^ Concentrations

J774A.1 cells were seeded on a 6-well cell culture plate at a density of 1 × 10^6^ cells/well and were subsequently treated with 0.5μg/mL of rPLO, rPLO W497F or rPLO D238R at 37°C for 30 min. The concentrations of intracellular K^+^ were then determined using cellular potassium concentration quantitative test kit (chemical colorimetric) (HL70032.2, Shanghai Haling Biological Technology Co., LTD) according to the instructions of the manufacturer.

### Western Blot Assays

J774A.1 cells were seeded on a 6-well cell culture plate at a density of 2×10^6^ cells/well. The cells were allowed to grow overnight. Before the experiment, the growth medium (RPMI-1640 medium supplemented with 10% FBS and penicillin–streptomycin) was replaced with RPMI-1640 medium without FBS and antibiotics. The cells were treated with bacteria, recombinant proteins, and/or chemicals (chemicals were added 1 h before the treatment with recombinant proteins). Then, the cells were collected and lysed in western-IP lysis buffer (P0013, Beyotime) on ice for 15 min. Undissolved fractions were removed by centrifugation (12000×*g*) at 4°C for 10 min. BCA assay was used to determine the protein concentration of the samples. The protein (30 μg) of each sample was separated by sodium dodecyl sulfate–polyacrylamide gel electrophoresis and then transferred to nitrocellulose (NC) membrane. The NC membrane was blocked with 5% skim milk at room temperature for 1 h. The membrane was incubated with primary antibody (1:1000 diluted) at 4°C overnight. Then, horseradish peroxidase-conjugated secondary antibody (1:5000 diluted) was incubated with the membrane at room temperature for 1 h. A BeyoECL Star kit (P0018AS, Beyotime) was used for detection.

To determine the expression of NLRP3 in J774A.1 cells, the cells were transduced with pooled Nlrp3 siRNA/shRNA/RNAi lentivirus (mouse) (iV043412, Abm Inc.) or scrambled siRNA GFP lentivirus (LVP015-G, Abm Inc.). The cells that were positively transduced with nlrp3 siRNA or control siRNA were selected with puromycin. The cells were primed with LPS (1 μg/mL) for 4 h and then treated with rPLO (0.5 μg/mL) for 0.5 h. The expression of NLRP3 was determined by western blot.

### Enzyme-Linked Immunosorbent Assay (ELISA)

ELISA kit (Cloud-Clone Corp) was used to detect the content of IL-1β (SEA563Mu) in the cell culture supernatant.

### Statistical Analysis

Statistical analysis was carried out according to the statistical methods indicated in the figure legends (Prism, GraphPad software). *p*<0.05 was considered significant (*p<0.05, **p<0.01, ***p<0.001, ****p<0.001).

## Results

### *T. pyogenes* Induced GSDMD Cleavage in J774A.1 Cells

**Figure 1:**
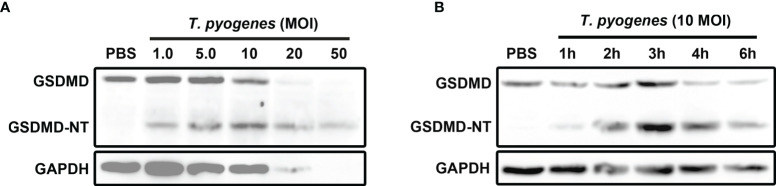
*T. pyogenes* treatment induced GSDMD cleavage in murine macrophages. **(A)** J774A.1 cells were treated with 1.0, 5.0, 10, 20 and 50 MOI of *T. pyogenes*, respectively, for 4 h. The blot shows the cleavage of GSDMD. **(B)** J774A.1 cells were treated with 10 MOI of *T. pyogenes* for 1 to 6 h. The blot shows the cleavage of GSDMD. The western blot assays were performed at least twice.

To determine the effect of *T. pyogenes* on pyroptosis, J774A.1 cells were incubated with *T. pyogenes*. As shown in **Figure 1A**, *T. pyogenes* treatment resulted in GSDMD cleavage in J774A.1 cells. Since cleavage of GSDMD is considered the core event in pyroptosis, the ability of *T. pyogenes* to induce pyroptosis in macrophages was confirmed. *T. pyogenes* at 1 multiplicity of infection (MOI) was sufficient to induce GSDMD cleavage within 4 h, and 10 MOI *T. pyogenes* showed the highest efficiency in causing GSDMD cleavage within 4 h. Moreover, 20 and 50 MOI *T. pyogenes* caused severe lysis of J774A.1 cells, which may interfere with the judgment of experimental results. Therefore, *T. pyogenes* at 10 MOI was used to treat cells in the following experiments.

Ten MOI *T. pyogenes* could induce GSDMD cleavage in J774A.1 cells within 1 h (**Figure 1B**). In these cells, GSDMD cleavage reached the peak within 3 h (**Figure 1B**).

### The Cholesterol-Sensitive Component(s) of *T. pyogenes* Is/Are Responsible for the GSDMD Cleavage Induced by *T. pyogenes* in J774A.1 Cells

**Figure 2:**
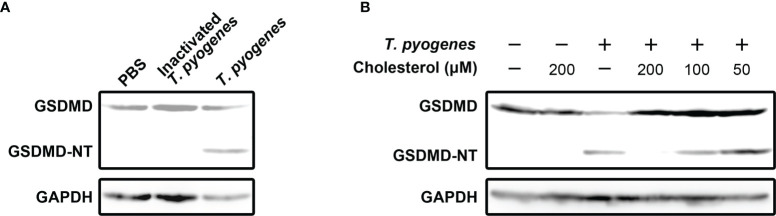
Cholesterol-sensitive substance(s) secreted by *T. pyogenes* induced GSDMD cleavage in murine macrophages. **(A)** J774A.1 cells were treated with 10 MOI *T. pyogenes* and inactivated *T. pyogenes* (about 2×10^8^ inactivated bacterial cells/well), respectively, for 1 h. The blot shows the cleavage of GSDMD. **(B)** 10 MOI *T. pyogenes* was used to treat J774A.1 cells concurrently with 50, 100 or 200 μM cholesterol. The blot shows the cleavage of GSDMD. The western blot assays were performed at least twice.

To identify the bacterial component(s) that play(s) (a) role(s) in inducing pyroptosis, formaldehyde-inactivated *T. pyogenes* and cholesterol were recruited into the current study. As seen in **Figure 2A**, formaldehyde-inactivated *T. pyogenes* could not cause GSDMD cleavage in J774A.1 cells, which indicates that the component(s) of *T. pyogenes* that caused GSDMD cleavage is/are not present in bacterial cells. We also found that the GSDMD cleavage induced by 10 MOI *T. pyogenes* was completely abolished by cholesterol (200 μM) (**Figure 2B**), suggesting that the bacterial component(s) responsible for *T. pyogenes* induced pyroptosis is/are sensitive to cholesterol.

### Preparation and Characterization of the rPLO Mutants

**Figure 3:**
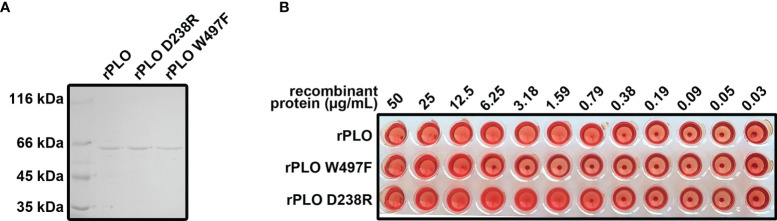
Preparation and characterization of rPLO and the mutants. **(A)** Purified rPLO, rPLO D238R and rPLO W497F were analyzed by SDS-PAGE. **(B)** Hemolysis assay of rPLO, rPLO D238R and rPLO W497F. Sheep red blood cells were mixed with serially diluted recombinant proteins. The mixtures were incubated at 37°C for 30 min. The smallest concentration of rPLO and rPLO D238R to cause complete hemolysis of sheep red blood cells were 0.79 μg/mL, 1.59 μg/mL and 12.5 μg/mL, respectively. The hemolysis assay was performed at least three times.

PLO is a cholesterol dependent cytolysin and is secreted by *T. pyogenes*. The features of PLO are consistent with the features of speculated components related to *T. pyogenes* induced pyroptosis. Therefore, rPLO and the two mutants (rPLO W497F, and rPLO D238R) were prepared (**Figure 3A**). The hemolytic activity of the recombinant proteins was measured. rPLO caused complete hemolysis in a sRBC system at a concentration as low as 0.79 µg/mL; rPLO D238R caused complete hemolysis at concentrations higher than 1.59 µg/mL; and rPLO W497F showed hemolytic activity at concentrations higher than 12.5 μg/mL (**Figure 3B**). The results indicate that the hemolysis activity of rPLO mutants was impaired.

Cell membrane binding assay showed that rPLO and rPLO D238R bound cell membrane with similar efficiency, whereas rPLO W497F had impaired cell membrane binding ability (**Supplementary Figure 1A**). Pores formed by rPLO could be observed by transmission electron microscope, whereas rPLO D238R formed incomplete pores in cell membrane (**Supplementary Figure 1B**). As described in previous works (8, 21), the D238R mutation affects the oligomerization of PLO monomers, while the W497F mutation impairs the cholesterol binding ability of PLO through causing changes in the conserved undecapeptide sequence.

### PLO Induced GSDMD Cleavage in J774A.1 Cells

**Figure 4:**
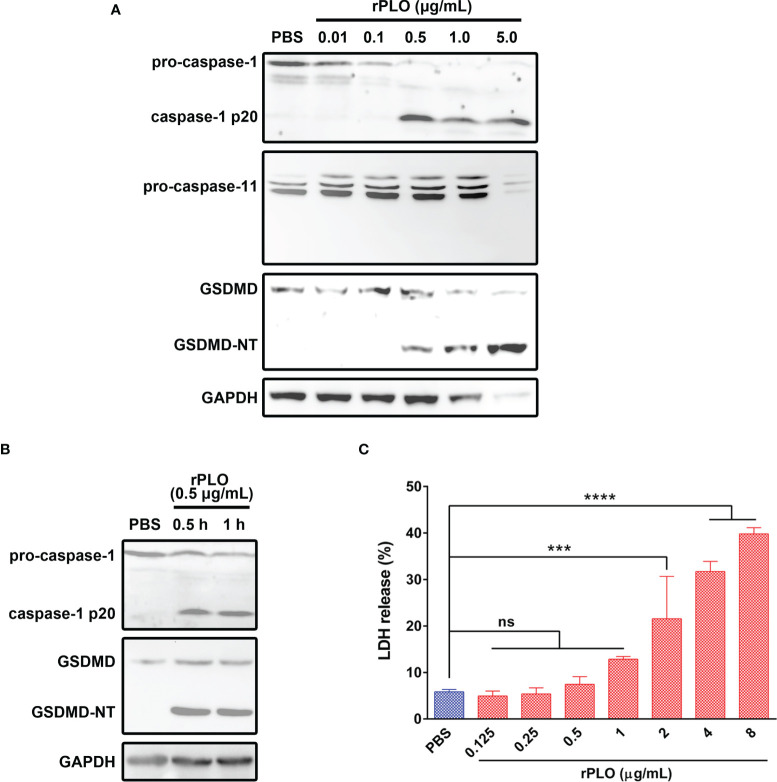
PLO induced caspase-1 activation and GSDMD cleavage in murine macrophages. **(A)** J774A.1 cells were treated with 0.01, 0.1, 0.5, 1.0 and 5.0 μg/mL rPLO, respectively, for 1 h. The blots showed the caspase-1 activation and GSDMD cleavage induced by rPLO. 0.5, 1.0 and 5.0 μg/mL rPLO could induce caspase-1 activation and GSDMD cleavage but could not induce caspase-11 activation. **(B)** J774A.1 cells were treated with 0.5 μg/mL rPLO for 0.5 h or 1 h. The blot showed the caspase-1 activation and GSDMD cleavage occurred within 0.5 h in the treated cells. **(C)** J774A.1 cells were treated with 0.125, 0.25, 0.5, 1.0, 2.0, 4.0 and 8.0 μg/mL rPLO, respectively, for 0.5 h. The LDH released from the cells was measured. The data was presented as the percentage of maximum LDH release of the cells. One-way ANOVA test was used to analyze the data (ns, no significance; ***p < 0.001; ****p < 0.0001). The western blot assays were performed at least twice. LDH release assay was technically repeated three times.

To determine whether PLO can induce pyroptosis of J774A.1 cells, the cells were incubated with rPLO. As seen in **Figure 4A**, rPLO at a concentration of not less than 0.5 μg/mL caused caspase-1 activation and GSDMD cleavage in J774A.1 cells within 1 h but did not cause caspase-11 activation. However, the blot of GAPDH showed that 1 and 5 μg/mL rPLO caused substantial cell lysis within 1 h. Therefore, 0.5 μg/mL was used as the working concentration of rPLO in the following experiments.

The results in **Figure 4B** show that 0.5 μg/mL rPLO caused caspase-1 activation and GSDMD cleavage within 0.5 h. However, 0.5 μg/mL rPLO treatment did not cause a remarkable increase in the LDH release of the cells (**Figure 4C**). This result means that within 0.5 h, the cells treated with 0.5 μg/mL rPLO are in the process of pyroptosis but not die due to pyroptosis.

### K^+^/NLRP3/Caspase-1 Pathway Is Responsible for PLO-Induced GSDMD Cleavage in J774A.1 Cells

Since caspase-1 activation is closely related to NLRP3 inflammasome (22), the current study focused on the activation mechanisms of the NLRP3 inflammasome. The western blot assay showed that J774A.1 cells constitutively express NLRP3 molecules (**Supplementary Figure 2**). RNA interference techniques were used to determine the accuracy of the western blot assay. The result showed that the NLRP3-specific siRNA, but not the control siRNA, significantly down-regulated the expression of the protein. Therefore, the bands on the blot indeed represent NLRP3 molecules. LPS priming and rPLO treatment did not significantly affect the expression of NLRP3 molecule in J774A.1 cells. Thus, in the current study, cells were not primed with LPS for NLRP3 expression.

**Figure 5:**
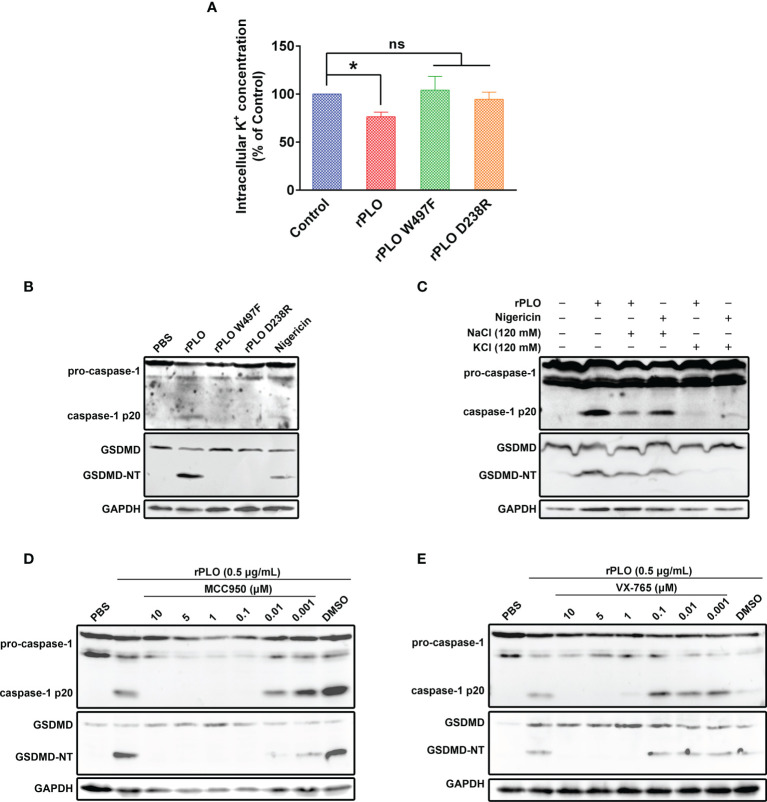
Determination of the role of K^+^/NLRP3/caspase-1 pathway in PLO-induced GSDMD cleavage. **(A)** J774A.1 cells were treated with 0.5 μg/mL rPLO, rPLO D238R and rPLO W497F, respectively, for 0.5 h. The intracellular K^+^ concentration of the cells was measured. The result was presented as a percentage of intracellular K^+^ concentration of the untreated cells. One-way ANOVA test was used to analyze the data (ns, no significance; *p <0.05). The experiment was technically repeated three times. **(B)** J774A.1 cells were treated with 0.5 μg/mL rPLO, rPLO D238R and rPLO W497F, respectively, for 0.5 h. Nigericin (5 mM) was used as a positive control. The blots show the caspase-1 activation and GSDMD cleavage. **(C)** J774A.1 cells were treated with 0.5 μg/mL rPLO and 120 mM KCl or NaCl for 0.5 h. The blots show the caspase-1 activation and GSDMD cleavage. **(D, E)** J774A.1 cells were treated with 0.5 μg/mL rPLO and different concentration of chemical inhibitors (MCC950 is a NLRP3 inhibitor; VX-765 is a caspase-1 inhibitor). The blots show the caspase-1 activation and GSDMD cleavage. The western blot assays were performed at least twice.

Since K^+^ efflux is a common trigger of NLRP3 inflammasome activation by bacterial toxins (22), the following work sought to find out the relationship between cellular K^+^ and rPLO. The intracellular K^+^ concentration of the recombinant proteins treated J774A.1 cells was first measured. As seen in **Figure 5A**, rPLO treatment resulted in significant K^+^ loss of the cells, while the two mutants did not cause significant K^+^ loss in 30 min. The results in **Figure 5B** show that the two rPLO mutants failed to induce caspase-1 activation and GSDMD cleavage in J774A.1 cells. The above results indicate that the pore-forming activity of PLO is necessary to induce intracellular K^+^ loss, caspase-1 activation and GSDMD cleavage.

The well-documented K^+^ ionophore, nigericin, also induced caspase-1 activation and GSDMD cleavage (**Figure 5B**), which indicates that K^+^ may play a key role in caspase-1 activation and GSDMD cleavage in J774A.1 cells. Therefore, in the next experiment, cells were co-treated with rPLO and 120 mM KCl or NaCl. The results showed that 120 mM KCl, but not 120 mM NaCl, completely abolished the caspase-1 activation and GSDMD cleavage induced by rPLO or nigericin (**Figure 5C**). The results indicate that K^+^ is related to the caspase-1 activation and GSDMD cleavage induced by rPLO in J774A.1 cells.

The involvement of NLRP3 inflammasomes in rPLO-induced caspase-1 activation and GSDMD cleavage was determined by using a specific NLRP3 inhibitor named MCC950. The results showed that 0.1 μM MCC950 completely abolished the caspase-1 activation and GSDMD cleavage induced by rPLO (**Figure 5D**). The results indicate that NLRP3 inflammasome is involved in rPLO-induced GSDMD cleavage.

Caspase-1 is one of the proteases that cleave GSDMD (11). Therefore, the caspase-1 inhibitor, VX-765, was used to determine whether caspase-1 mediates GSDMD cleavage in rPLO-treated cells. The results showed that 1 μM VX-765 completely inhibited the GSDMD cleavage induced by rPLO treatment (**Figure 5E**). The results indicate that caspase-1 plays a major role in mediating GSDMD cleavage.

Taken these results together, K^+^/NLRP3/caspase-1 pathway is responsible for PLO-induced GSDMD cleavage in J774A.1 cells.

### NSA but Not Nec-1 Inhibited rPLO-Induced GSDMD Cleavage

**Figure 6:**
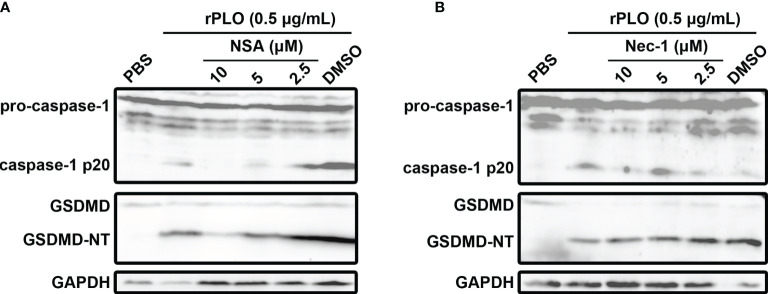
The effect of necrosulfonamide and necrostatin-1 on PLO-induced caspase-1 activation and GSDMD cleavage. **(A)** J774A.1 cells were treated with 0.5 μg/mL rPLO and different concentration of necrosulfonamide (NSA) (added 1 h before rPLO treatment) for 0.5 h. The blots show the caspase-1 activation and GSDMD cleavage. **(B)** J774A.1 cells were treated with 0.5 μg/mL rPLO and different concentration of necrostatin-1 (Nec-1) (added 1 h before rPLO treatment) for 0.5 h. The blots show the caspase-1 activation and GSDMD cleavage. The western blot assays were performed at least twice.

To rule out the possibility that PLO induced GSDMD cleavage is a downstream event of cell necroptosis, NSA and Nec-1, the two chemicals that can inhibit necroptosis (23), were used in the current study. Our results showed that 10 and 5 μM NSA substantially inhibited rPLO-induced caspase-1 activation and GSDMD cleavage (**Figure 6A**), whereas Nec-1 did not show this ability (**Figure 6B**).

### Chemical Inhibitors Attenuated rPLO-Induced Cellular LDH Release

**Figure 7:**
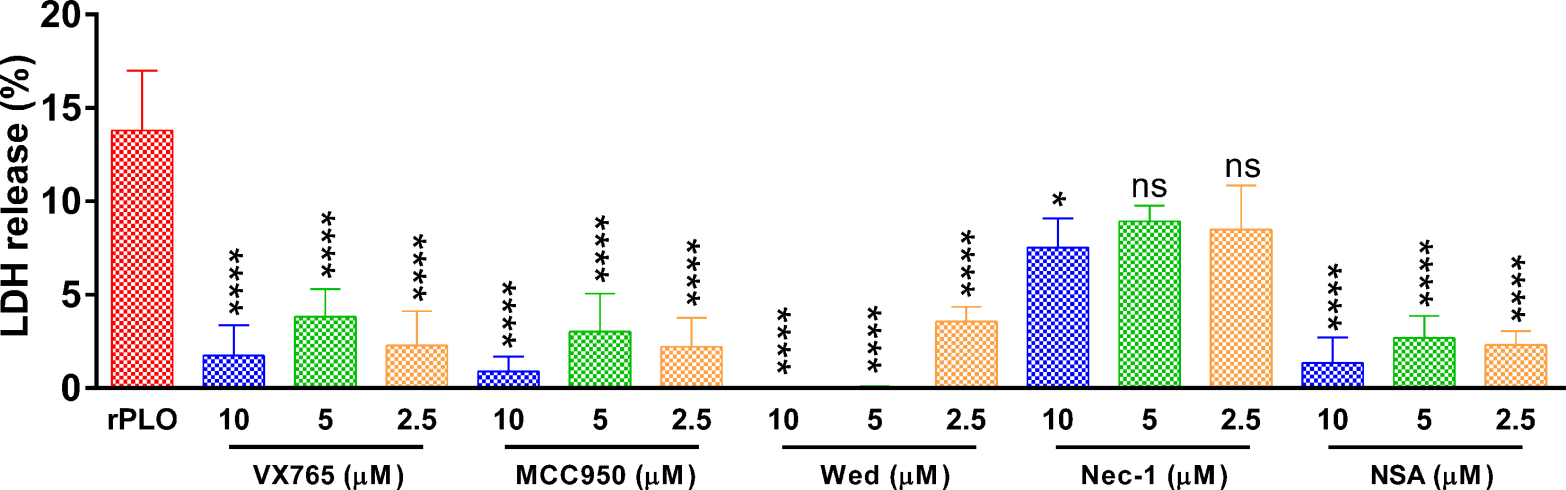
The effect of chemical inhibitors on PLO-induced LDH release from J774A.1 cells. J774A.1 cells were treated with 0.5 μg/mL rPLO and different concentration of chemical inhibitors (Wed, Wedelolactone; Nec-1, necrostatin-1; NSA, necrosulfonamide) for 1 h. The LDH released from the cells was measured. The data was presented as the percentage of maximum LDH release of the cells. One-way ANOVA test was used to analyze the data (ns, no significance; *p < 0.05; ****p < 0.0001). LDH release assays were technically repeated three times.

**Figure 7** shows that at 1 h, the inhibitors, except for Nec-1, efficiently inhibited rPLO-induced cellular LDH release (**Figure 7**). Since it is impossible that all inhibitors can react directly with rPLO, the inhibition of cellular LDH release by these inhibitors should be mainly attributed to their inhibitory effect on pyroptotic lysis, implying that 0.5 μg/mL rPLO by itself did not cause severe cell membrane damage. Thus, severe cell membrane damage was caused by GSDMD cleavage induced by rPLO treatment.

### Chemical Inhibitors Significantly Inhibited *T. pyogenes*/rPLO-Induced IL-1β Production in J774A.1 Cells

**Figure 8:**
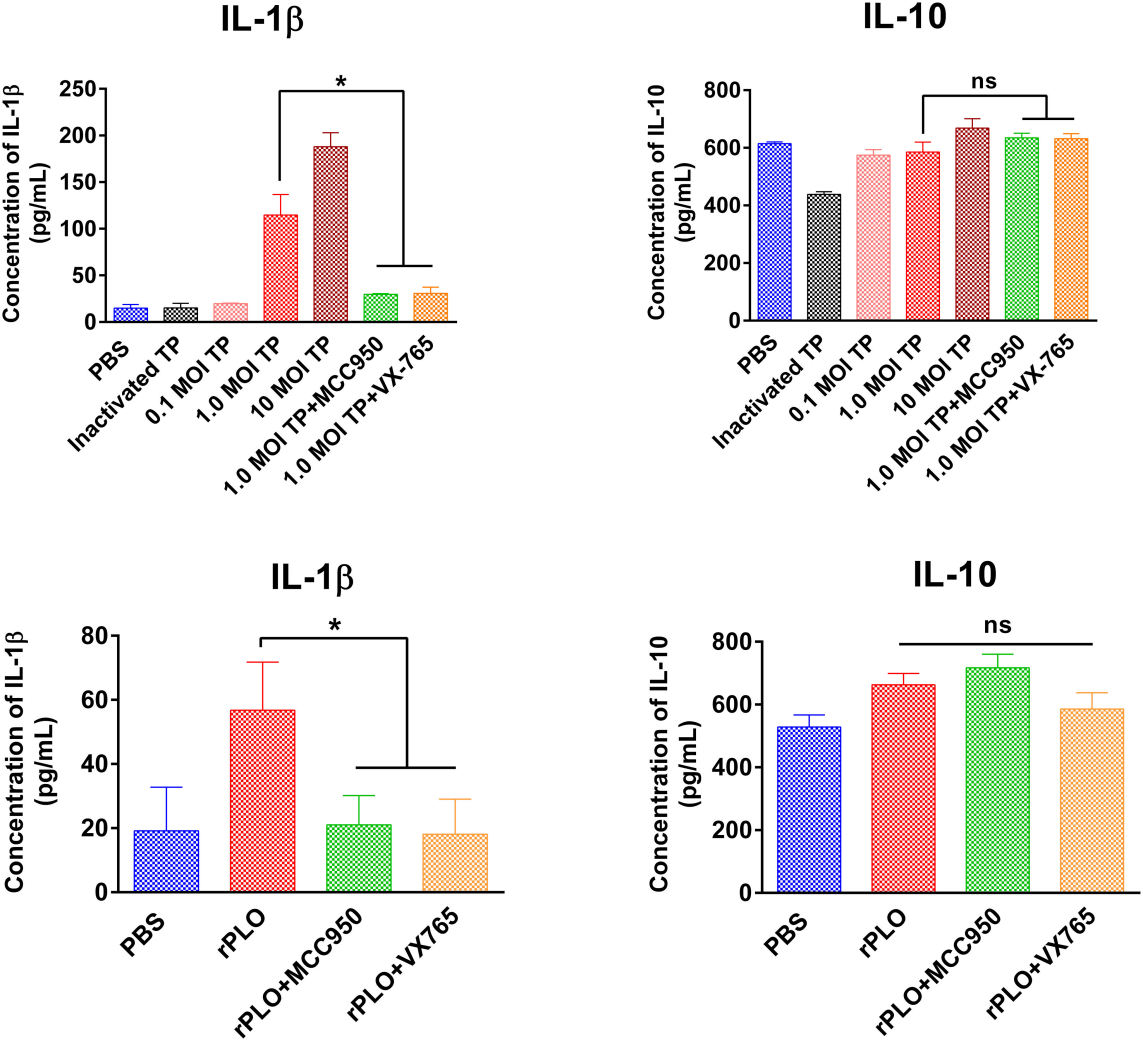
The effect of MCC950 and VX-765 on *T. pyogenes* and PLO-induced IL-1β and IL-10 release from J774A.1 cells. **(A)** J774A.1 cells were treated with 0.1, 1, and 10 MOI *T. pyogenes* (TP), respectively, for 3 h. MCC950 and VX-765 were used to treat cells concurrently with 1 MOI of TP. The content of IL-1β (left panel) and IL-10 (right panel) in the culture supernatant was measured. **(B)** J774A.1 cells were treated with 0.5 μg/mL rPLO and MCC950 or VX-765 for 1 h. The content of IL-1β (left panel) and IL-10 (right panel) in the culture supernatant was measured. One-way ANOVA test was used to analyze the data. (ns, no significance; *p < 0.05). The assays were technically repeated three times.

J774A.1 cells were treated with 0.1, 1.0, and 10 MOI *T. pyogenes* for 3 h. Treatment with 1.0 and 10 MOI *T. pyogenes* for 3 h resulted in a remarkable increase in the IL-1β content of the cell culture supernatant, whereas 2.5 μM MCC950 and VX-765 markedly inhibited the increase in IL-1β production caused by 1.0 MOI *T. pyogenes* (**Figure 8A**). The IL-10 production induced by 1.0 MOI *T. pyogenes* was not remarkably affected by these two inhibitors (**Figure 8A**).

Treatment with 0.5 μg/mL rPLO for 1 h led to a considerable increase in IL-1β production and treatment with 2.5 μM MCC950 and VX-765 substantially inhibited the increase in IL-1β production caused by rPLO (**Figure 8B**). IL-10 production was not remarkably affected by these inhibitors (**Figure 8B**).

## Discussion

Macrophages are ubiquitous in tissues and are an indispensable member of the host’s first-line immune defense against microbial infections (24). Gonzalez-Juarbe et al. (25) reported the specific depletion of alveolar macrophages in mice infected with *Serratia marcescens* and determined that the rapid death of macrophages was caused by the PFT secreted by the bacteria. In a following work, they proposed that killing macrophages is the common key first step for PFT-producing pathogens to establish infection (9). These reports indicate that the contact between macrophages and pathogens that produce PFT is common. Therefore, macrophages need to be used in the study of the pathogenesis mechanism of *T. pyogenes*.

*T. pyogenes* infection can cause inflammation in different organs or tissues of the host (8, 26). *T. pyogenes* can promote the expression of IL-1β, an important proinflammatory cytokine, in experimentally infected bovine (27), bovine endometrial epithelial cells (28), and mouse macrophages (29). These studies indicate that *T. pyogenes* may aggravate inflammation by inducing IL-1β expression in the host. The expression of active IL-1β requires a two-step process. In the first step, pro-IL-1β is cleaved by proteases (such as caspase-1). In the second step, the active form of IL-1β is released through pores in the plasma membrane or after the cell ruptures (14). Affecting any of these two steps may result in changes in the IL-1β expression profile. In the current study, we first found that *T. pyogenes* caused the cleavage of GSDMD (one of the executioners of pyroptosis) in mouse macrophages (**Figure 1**). The N-terminal fragment of GSDMD can mediate the release of mature IL-1β by forming pores in the plasma membrane or causing cell pyrolysis (11). We speculated that the tendency of *T. pyogenes* to promote IL-1β expression and aggravate the inflammatory response may be due to its ability to cause GSDMD cleavage. Therefore, the focus of the following work was to reveal the underlying mechanism of how *T. pyogenes* causes GSDMD cleavage.

Cholesterol inhibited the GSDMD cleavage caused by *T. pyogenes* (**Figure 2B**). This result means that the *T. pyogenes* component responsible for inducing GSDMD cleavage is sensitive to cholesterol. It has been reported that PLO of *T. pyogenes* can bind to cholesterol microcrystals (30). Therefore, we speculated that neutralization of the binding activity of PLO to cholesterol-containing plasma membranes by cholesterol is the reason why cholesterol inhibited the GSDMD cleavage induced by *T. pyogenes*. Thus, cells were treated with purified rPLO. **Figure 4** shows that rPLO induced GSDMD cleavage in J774A.1 cells, which supports our speculation.

The cleavage of GSDMD in rPLO-treated macrophage suggests that PFT-treated cells may undergo pyroptosis. Besides triggering pyroptosis, Gonzalez-Juarbe et al. (9), Kitur et al. (31), and Gilley et al. (32) reported that PFTs, including PLY, can kill macrophages by triggering necroptosis. PFTs also induces apoptosis in different type of cells (33–35). These results suggest a complex role for PFTs in inducing cell death.

In mouse cells, GSDMD can be cleaved by caspase-1 (canonical pathway) or caspase-11 (noncanonical pathway) (11, 12). The result in **Figure 4A** shows that 0.5 μg/mL rPLO is sufficient to activate caspase-1 in J774A.1 cells within 1 h. The dynamic characteristics of caspase-1 activation and GSDMD cleavage in J774A.1 cells caused by rPLO are consistent. VX-765, a specific caspase-1 inhibitor, completely abolished the rPLO-induced GSDMD cleavage at the concentration of 1 μM (**Figure 5E**). The activation of caspase-11 requires the presence of lipopolysaccharide (LPS) in the cytoplasm (12). LPS is a component of the cell wall of Gram-negative bacteria. *T. pyogenes* is a Gram-positive bacterium (*T. pyogenes* cells have no LPS); therefore, theoretically, *T. pyogenes* cannot directly activate mouse caspase-11. Moreover, we tested whether caspase-11 was activated in rPLO-treated cells to exclude the possibility that the residual LPS in purified rPLO resulted in GSDMD cleavage by activating caspase-11. The result showed that rPLO treatment did not activate caspase-11 in J774A.1 cells (**Figure 4A**). These data indicate that caspase-1 is the main executioner of GSDMD cleavage in PLO-treated cells.

Unlike rPLO, the two rPLO mutants (rPLO W497F and rPLO D238R) failed to induce caspase-1 activation and GSDMD cleavage (**Figure 5A**). This result indicates that the binding and pore-forming activity of PLO is necessary for inducing caspase-1 activation and GSDMD cleavage.

Plasma membrane damage usually leads to the uncontrolled movement of ions across the membrane (36). The loss of K^+^ in cells is a common trigger for caspase-1 activation through the NLRP3 inflammasome pathway (22). The result showed that rPLO treatment resulted in a significant loss of intracellular K^+^ in J774A.1 cells (**Figure 5A**). Therefore, we further examined the effect of high-concentration extracellular K^+^ on rPLO-induced caspase-1 activation and GSDMD cleavage. The result showed that 120 mM KCl, but not 120 mM NaCl, completely abolished rPLO-induced caspase-1 activation and GSDMD cleavage in J774A.1 cells (**Figure 5B**). The data indicate that K^+^ plays a role in the process of PLO-induced caspase-1 activation and GSDMD cleavage. Interestingly, 120 mM NaCl partially inhibited rPLO-induced caspase-1 activation but did not affect nigericin-induced caspase-1 activation. The difference may be attributed to the different mechanisms used by nigericin and PLO to induce K^+^ efflux. Nigericin is a specific K^+^ transporter that can transport only intracellular K^+^ to the extracellular environment. By contrast, PLO molecules form pores in the cell membrane, which may cause nonspecific ion movement across the cell membrane. Thus, chloride (Cl^−^) may also flow out through the pores formed by PLO molecules. The decrease in intracellular Cl^−^ concentration can also lead to the activation of NLRP3 inflammasomes and caspase-1 (37). Adding NaCl to the medium to form an extracellular environment with a high concentration of Cl^−^ may inhibit the loss of intracellular Cl^−^, which partially inhibits rPLO-induced caspase-1 activation and GSDMD cleavage. This result indicates that PLO induces caspase-1 activation and GSDMD cleavage in a more complicated manner.

MCC950 (0.01 μM), a well-documented NLRP3 inflammasome inhibitor, completely inhibited rPLO-induced caspase-1 activation and GSDMD cleavage (**Figure 5D**). The result indicates that NLRP3 inflammasome plays a key role in the process of PLO-induced caspase-1 activation and GSDMD cleavage.

These results suggest that the GSDMD cleavage in PLO-treated J774A.1 cells may be attributed to the activation of the NLRP3/caspase-1 pathway by PLO through forming pores in the plasma membrane and causing the loss of intracellular K^+^.

**Figure 9:**
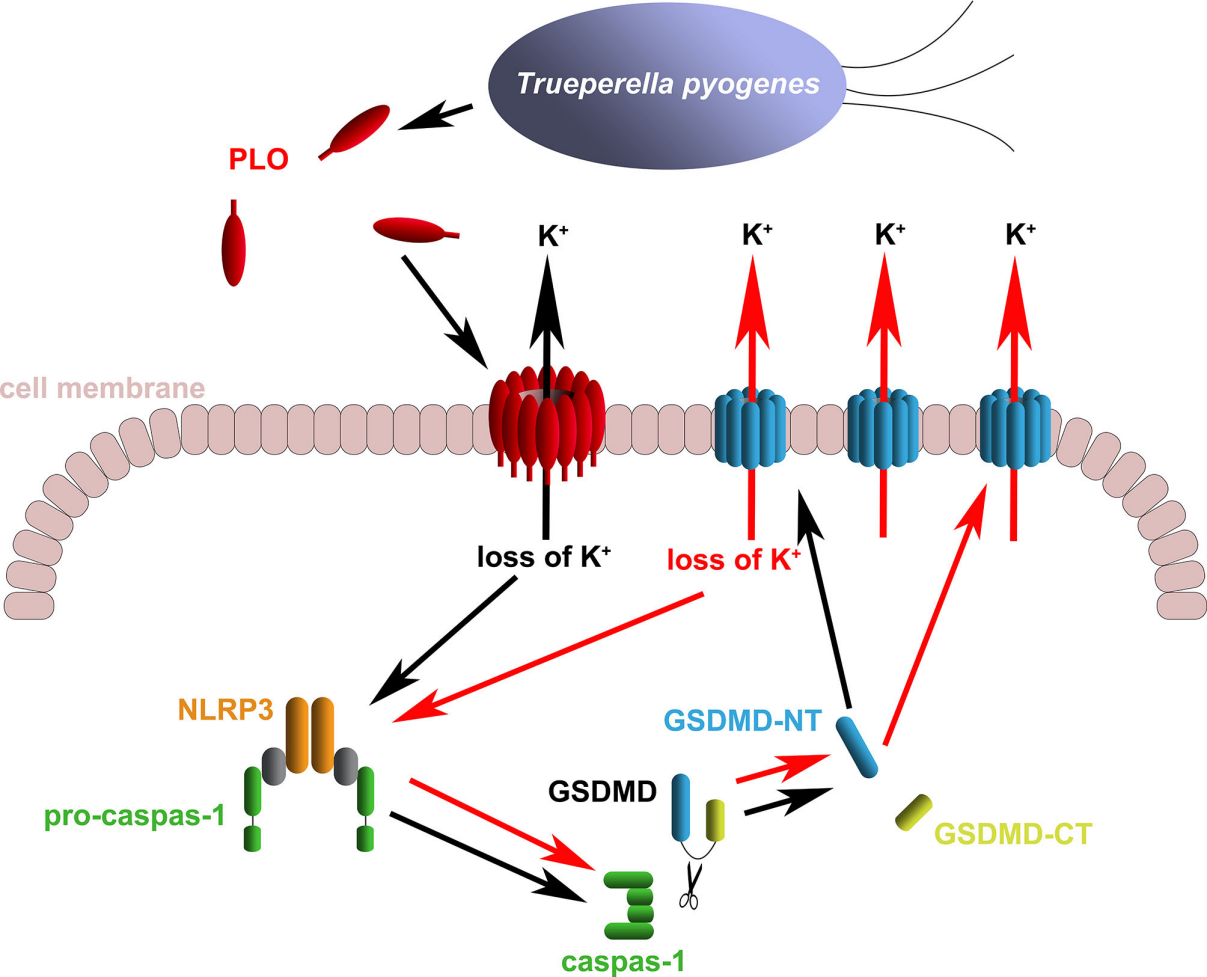
Diagram of the mechanism of *T. pyogenes* causing GSDMD cleavage. The black arrows show the basic event caused by *T. pyogenes*. The red arrows show the positive feedback loop of GSDMD cleavage.

The above speculation seems rational. However, the activation of the NLRP3/caspase-1 pathway is a primary event induced by rPLO treatment or a downstream event to some primary events is still a mystery. Fortunately, some interesting results give us clues to the truth. Previous studies have suggested that activation of NLRP3/caspase-1 pathway may be a downstream event of necroptosis, as the necroptosis executioner MLKL can form pores in cell membrane and thereby resulting in loss of intracellular K^+^ (38, 39). In the current study, two necroptosis inhibitors, Nec-1 and NSA, were used to determine the role of necroptosis in PLO-induced caspase-1 activation and GSDMD cleavage. If necroptosis occurs in rPLO-treated J774A.1 cells and is an upstream event of pyroptosis, both NSA and Nec-1 would abolish rPLO-induced caspase-1 activation and GSDMD cleavage by inhibiting MLKL activation theoretically. Interestingly, NSA inhibited rPLO-induced caspase-1 activation and GSDMD cleavage, whereas Nec-1 did not show this ability (**Figure 6**). Nec-1 did not affect rPLO-induced caspase-1 activation and GSDMD cleavage indicates that rPLO did not induce necroptosis in J774A.1 cells (**Figure 6B**). For the experiment involving NAS, the result seems somewhat confusing (**Figure 6A**). However, a recent study showing that NSA can bind GSDMD directly to inhibit pyroptosis (40) would be helpful to explain the result of the experiment involving NSA. This would be another story where the secondary GSDMD cleavage is the downstream event of primary GSDMD cleavage. rPLO treatment induced limited primary activation of K^+^/NLRP3/caspase-1 pathway and GSDMD cleavage. K^+^ efflux through the primary GSDMD-NT pores led to substantial secondary activation of NLRP3/caspase-1 pathway and GSDMD cleavage (**Figure 9**). In this model, PLO only “ignites a small fire” and the cleavage products of GSDMD “make the fire more prosperous”. According to this model, NSA blocked the formation of primary GSDMD-NT pores, thereby weakening the secondary event and the further positive feedback loops.

In LDH release assay, MCC950, VX-765 and NSA significantly inhibited rPLO-induced LDH release from J774A.1 cells (**Figure 7**), which is in accordance with the results in **Figures 5D, E**, **6A**. These results together indicate that rPLO caused cell membrane damage mainly through inducing GSDMD cleavage. Wed, a widely used caspase-11 inhibitor, also inhibited rPLO-induced LDH release from J774A.1 cells. This does not indicate the involvement of caspase-11 in rPLO-induced pyroptosis, as rPLO treatment did not activate caspase-11 in J774A.1 cells (**Figure 4A**). The inhibition of rPLO-induced LDH release by Wed may be attributed to its inhibitory effect on NLRP3 inflammasome, as a recent study showed that Wed can promote NLRP3 phosphorylation, thereby blocking inflammasome activation and pyroptosis (41).

The effect of *T. pyogenes* on IL-1β and IL-10 production in J774A.1 cells was determined. According to the results, 1.0 MOI *T. pyogenes* resulted in increased IL-1β production after 3 h of treatment (**Figure 8A**). As shown in **Figure 1A**, 1.0 MOI *T. pyogenes* caused GSDMD cleavage, indicating that *T. pyogenes* may promote the release of IL-1β by leading to the formation GSDMD-NT pores in cell membranes. The inhibition of MCC950 and VX-765 on *T. pyogenes*-induced increase in IL-1β production (**Figure 8A**) suggests that *T. pyogenes*-induced increase of IL-1β production in the cells is related to the NLRP3/caspase-1/GSDMD axis. The inhibition of this axis may hinder the release of IL-1β from the cells.

*T. pyogenes*-induced increase in IL-1β production in J774A.1 cells is at least partially attributable to PLO-induced IL-1β release, as rPLO treatment resulted in GSDMD cleavage (**Figures 4**–**6**) and an increase in IL-1β production (**Figure 8B**). Inhibition of rPLO-induced increase in IL-1β production by MCC950 and VX-765 confirmed the speculation (**Figure 8B**), as both inhibitors could abolish rPLO-induced GSDMD cleavage (**Figures 5D, E**).

In conclusion, our works uncovered a mechanism by which *T. pyogenes* affects the expression of proinflammatory cytokines. We determined that *T. pyogenes* can cause GSDMD cleavage in macrophages by secreting PLO. PLO causes the loss of intracellular K^+^ by forming pores in the cell membrane. The loss of intracellular K^+^ activates the NLRP3 inflammasome/caspase-1 pathway, which leads to GSDMD cleavage. GSDMD-NT leads to further loss of intracellular K^+^, thereby forming a positive feedback loop for GSDMD cleavage. PLO may facilitate the release of IL-1β by causing GSDMD cleavage. These data provide important information for understanding the pathogenicity of *T. pyogenes*.

## Data Availability Statement

The original contributions presented in the study are included in the article/**Supplementary Material**. Further inquiries can be directed to the corresponding authors.

## Author Contributions

WZ designed the experiments, analyzed the results, supervised the work, and drafted the manuscript. HL performed most of the experiments. BW performed some of the western blot assays. BM and JW supervised the work and revised the manuscript. All authors contributed to the article and approved the submitted version.

## Funding

This work was supported by the University Nursing Program for Young Scholars with Creative Talents in Heilongjiang Province, grant number UNPYSCT-2017019 and “Academic Backbone” Project of Northeast Agricultural University, grant number 17XG09.

## Conflict of Interest

The authors declare that the research was conducted in the absence of any commercial or financial relationships that could be construed as a potential conflict of interest.

## Publisher’s Note

All claims expressed in this article are solely those of the authors and do not necessarily represent those of their affiliated organizations, or those of the publisher, the editors and the reviewers. Any product that may be evaluated in this article, or claim that may be made by its manufacturer, is not guaranteed or endorsed by the publisher.

## Supplementary Material

Supplementary Figure 1 Determination of the cell membrane binding and pore-forming ability of rPLO and the two mutants. **(A)** Sheep red blood cell membranes were incubated with the recombinant proteins at 37°C for 30 min. The mixtures were centrifuged, and the precipitates were collected. The precipitates were washed twice with PBS and dissolved in NaOH. The samples were subjected to western blot analysis using a monoclonal mouse anti-His tag antibody as primary antibody. **(B)** 40 μg of recombinant proteins was incubated with 900 μL 2% sheep red blood cells at 37°C for 30 min. The mixtures were centrifuged at 5000 r/min for 7 min at 4°C, and the supernatant was discarded. The precipitates were resuspended in 200 μL fresh PBS. Samples were stained with 1% (wt./vol.) uranyl acetate and observed using a Hitachi H-7650 electron microscope at an acceleration voltage of 100 kV. Scale bar=0.2μm. White arrows indicates the pores formed by rPLO or rPLO D238R.

Supplementary Figure 2 Determination of the expression of NLRP3 molecules in J774A.1. J774A.1 cells constitutively express NLRP3 molecules. LPS and rPLO stimulation did not significantly affect the expression of NLRP3 molecules.
