# Supplementary figures and images for: Pyolysin of Trueperella pyogenes Induces Pyroptosis and IL-1β Release in Murine Macrophages Through Potassium/NLRP3/Caspase-1/Gasdermin D Pathway
Source: Front Immunol. 2022 Mar 15;13:832458. doi: 10.3389/fimmu.2022.832458 (PMC8965163; doi:10.3389/fimmu.2022.832458)

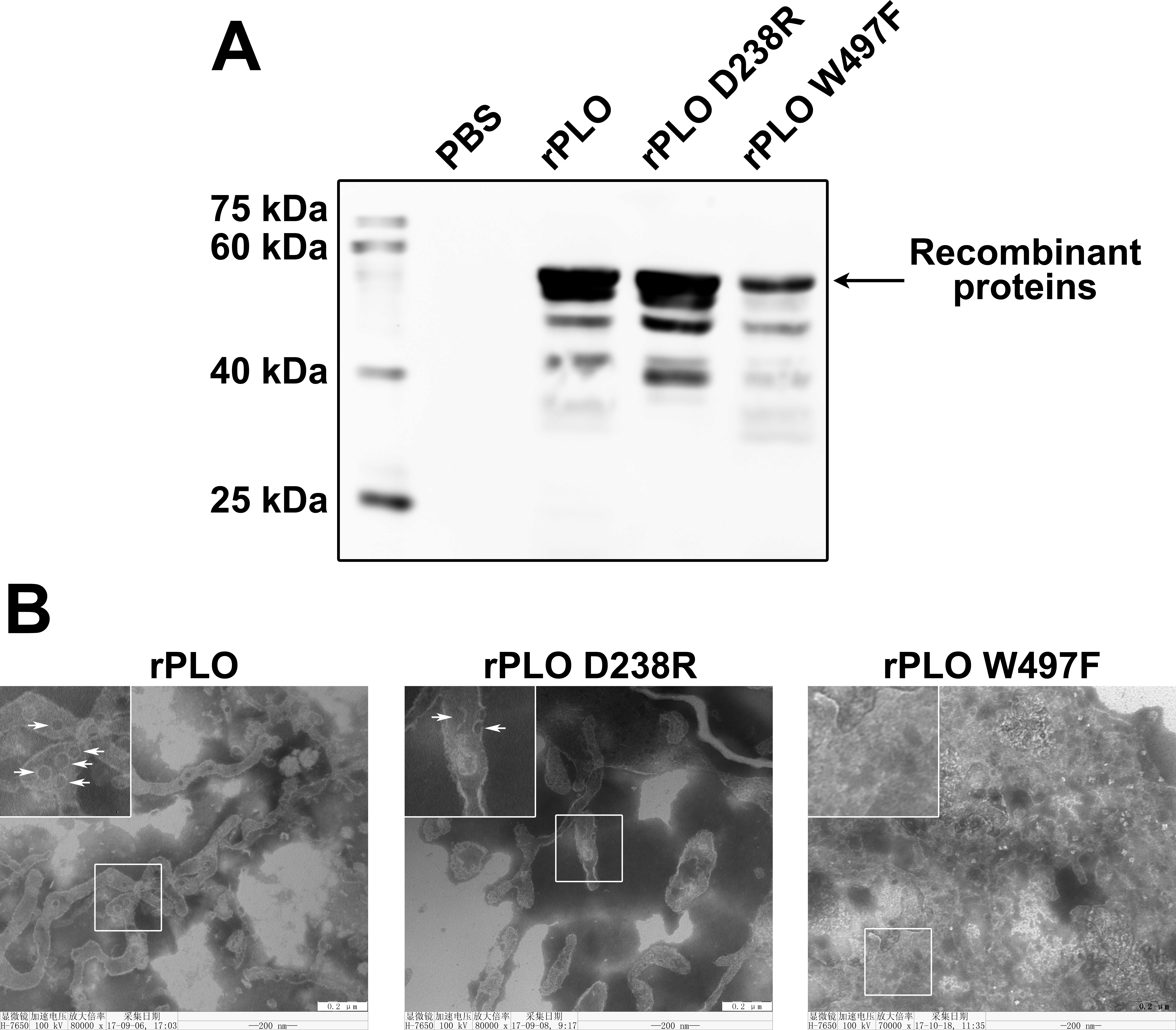

Supplement: Supplementary Figure 1 — Determination of the cell membrane binding and pore-forming ability of rPLO and the two mutants. (A) Sheep red blood cell membranes were incubated with the recombinant proteins at 37°C for 30 min. The mixtures were centrifuged, and the precipitates were collected. The precipitates were washed twice with PBS and dissolved in NaOH. The samples were subjected to western blot analysis using a monoclonal mouse anti-His tag antibody as primary antibody. (B) 40 μg of recombinant proteins was incubated with 900 μL 2% sheep red blood cells at 37°C for 30 min. The mixtures were centrifuged at 5000 r/min for 7 min at 4°C, and the supernatant was discarded. The precipitates were resuspended in 200 μL fresh PBS. Samples were stained with 1% (wt./vol.) uranyl acetate and observed using a Hitachi H-7650 electron microscope at an acceleration voltage of 100 kV. Scale bar=0.2μm. White arrows indicates the pores formed by rPLO or rPLO D238R. [file Image_1.tif]

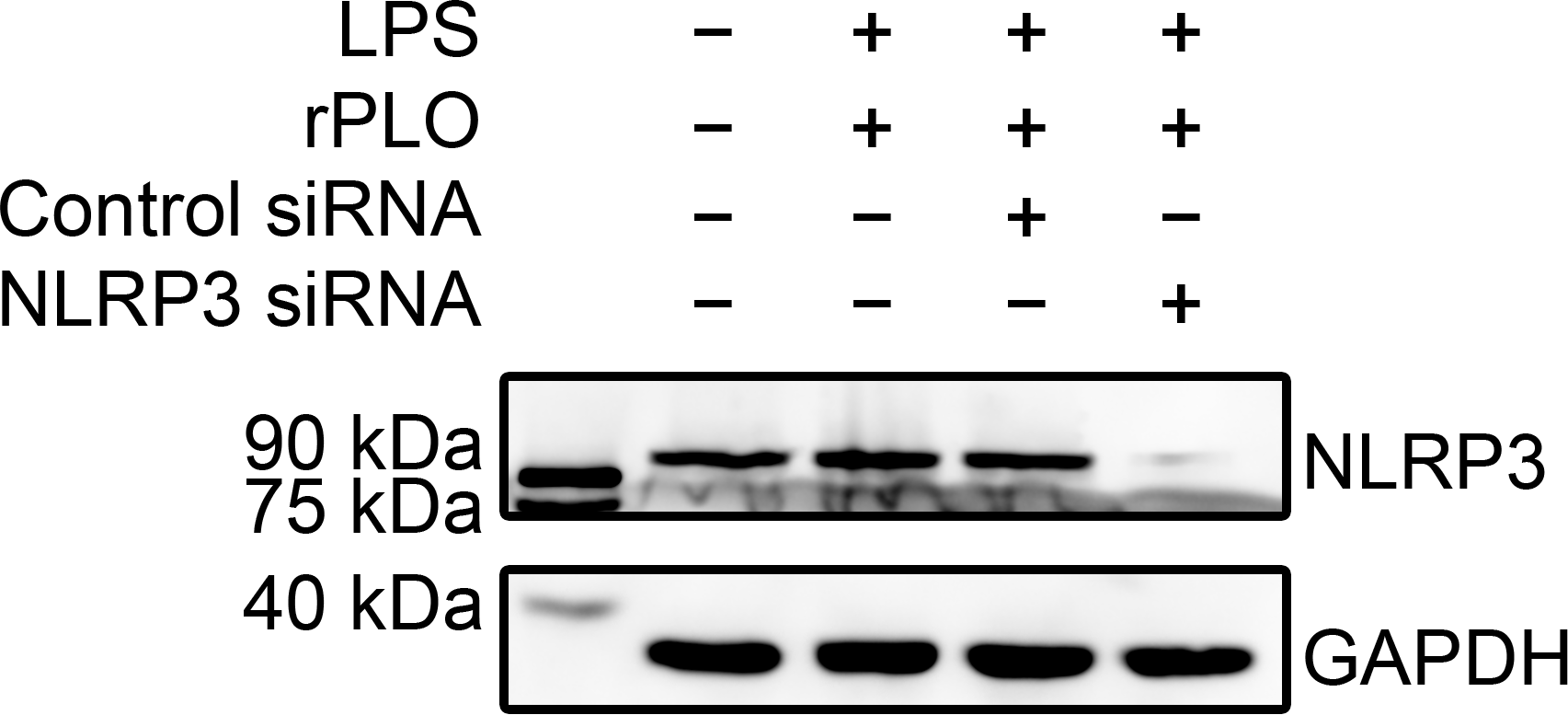

Supplement: Supplementary Figure 2 — Determination of the expression of NLRP3 molecules in J774A.1. J774A.1 cells constitutively express NLRP3 molecules. LPS and rPLO stimulation did not significantly affect the expression of NLRP3 molecules. [file Image_2.tif]
